# Our Voluntary Hospitals and Institutions

**Published:** 1915-12-18

**Authors:** 


### OUR VOLUNTARY HOSPITALS & INSTITUTIONS.

### What should they Publish about their Work and Needs?

*To the Editor of* THE HOSPITAL.

SIR,—Your article in last week's HOSPITAL raises once more the interesting question of the extent to which statistical information can be usefully carried in the hospital world.

It cannot be gainsaid that much information is still lacking which would enable a member of the public to compare the exact details of management in different institutions, but it has yet to be established that any generous donors are guided in their distribution of charity by such considerations.

In nearly seven years' secretarial work in a fairly large hospital I do not remember receiving a single inquiry from the outside public as to even the larger questions of management. Most of the great charities which minister to other needs than those of the sick publish far less information than is now provided in the hospital report, and some of the worst offenders seem to be the most prosperous.

The Uniform System of Accounts has enabled hospital officials, with their special knowledge of local conditions, to estimate pretty fairly the position of their own hospital in the principal items of expenditure, and this result has been of undoubted value, though achieved at considerable cost.

Such a departure as you suggest would involve largely increased expenditure upon management, with only a problematical advantage. A good case can be made for increasing the clerical staff of most hospitals, where an actual saving might be achieved—*i.e.*, in the department which deals with contracts, markets, and prices—but I beg you, Sir, to save us from a further multitude of figures designed to please a mythical member of the public who has never existed, who never will exist, and would have no money to give away if he did.—Yours faithfully,

ASSISTANT SECRETARY.

SOME FACTS ABOUT GIVING.

[It would be interesting to have the details which make up *the considerable cost* which "Assistant Secretary" claims has been entailed "in estimating pretty fairly the position of his hospital in the principal items of expenditure." There should be no such cost, considerable or otherwise, in a hospital properly organised on a business basis. Our valued correspondent holds unique views about the withholding of information from the subscribers and supporters of hospitals and benevolent institutions. The facts give a direct negative to his contention in the second paragraph of his letter. The circulation of accurate information and figures widely distributed through the King's Fund, and by the more businesslike and better managed hospitals, has contributed to the increase of the income of the voluntary hospitals in eighteen years from £2,528,000 in 1896 to £4,677,000 in 1913, or by 85 per cent. That increase would have been impossible had it not been for the work and labour expended upon the preparation and circulation of information resting on audited figures. This convincing information has enabled the public who may be interested to compare the exact details of management in different institutions and to cause the distribution of their charity to accomplish many things not previously possible to the givers. This development has been the main cause of the enormous growth in the income of the voluntary hospitals referred to. Further, the hospitals have had collectively a total surplus in money during the five years 1909-1913 of one and three-quarter million pounds, representing an average surplus of £350,000 per annum.

If "Assistant Secretary" were in responsible charge of a great hospital, and carried out in practice his theory of suppressing or not circulating much information of an interesting and perfectly reliable character, the funds of that hospital in the course of ten years would certainly, and properly, experience a marked decrease in the amount of money received from the public. The truth is a man may concentrate his whole energies upon the details of his work for his particular charity to the exclusion of the wider interests and facts upon which the development and enormous progress in the financial sense of our voluntary hospitals have depended increasingly during the last twenty, and especially during the last ten, years of their existence. He may so fall behind in method and apprehension. Those hospitals which give the fullest and frankest information, and circulate it most widely, will secure, as they deserve, an abundant harvest in their monetary return. Our correspondent has a special hospital, the work of which appeals more widely to the whole nation and Empire than probably any other in existence. It has further practically no competitors to diminish the yield, which experience proves can be continuously increased by the application of the right methods when backed by the results of the work it does so well. But "One swallow does not make a summer."—ED. THE HOSPITAL.]

### "The Population Problem."

*To the Editor of* THE HOSPITAL.

